# Coryza

**Published:** 1874-05

**Authors:** J. S. Prout


*THE MEDICAL RECORD—January,* 1874.

Treatment of Coryza.—*By J. S. Prout, M.D.*—Let me state that I have, in my own person, and with patients, often been able to arrest the disease in the course of an hour or less, by taking or giving large doses of the officinal tincture of the chloride of iron, twenty or thirty minims, as soon as possible after the cold is "caught." I generally find that in about half an hour there is a decided amelioration of the symptoms, which may be permanent, in which case I take or give no more of the tincture, or the improvement may pass off in two or three hours; in which case, the dose must be repeated. This may be required three or four times.

